# Simultaneous multislice steady‐state free precession myocardial perfusion with full left ventricular coverage and high resolution at 1.5 T

**DOI:** 10.1002/mrm.29229

**Published:** 2022-03-28

**Authors:** Sarah McElroy, Giulio Ferrazzi, Muhummad Sohaib Nazir, Carl Evans, Joana Ferreira, Filippo Bosio, Nabila Mughal, Karl P. Kunze, Radhouene Neji, Peter Speier, Daniel Stäb, Tevfik F. Ismail, Pier Giorgio Masci, Adriana D. M. Villa, Reza Razavi, Amedeo Chiribiri, Sébastien Roujol

**Affiliations:** ^1^ School of Biomedical Engineering and Imaging Sciences, Faculty of Life Sciences and Medicine King's College London London UK; ^2^ IRCCS San Camillo Hospital Venice Italy; ^3^ MR Research Collaborations, Siemens Healthcare Limited Frimley England UK; ^4^ Magnetic Resonance Siemens Healthcare GmbH Erlangen Germany; ^5^ MR Research Collaborations Siemens Healthcare Limited Melbourne Australia

**Keywords:** compressed sensing, myocardial perfusion, simultaneous multi‐slice

## Abstract

**Purpose:**

To implement and evaluate a simultaneous multi‐slice balanced SSFP (SMS‐bSSFP) perfusion sequence and compressed sensing reconstruction for cardiac MR perfusion imaging with full left ventricular (LV) coverage (nine slices/heartbeat) and high spatial resolution (1.4 × 1.4 mm^2^) at 1.5T.

**Methods:**

A preliminary study was performed to evaluate the performance of blipped controlled aliasing in parallel imaging (CAIPI) and RF‐CAIPI with gradient‐controlled local Larmor adjustment (GC‐LOLA) in the presence of fat. A nine‐slice SMS‐bSSFP sequence using RF‐CAIPI with GC‐LOLA with high spatial resolution (1.4 × 1.4 mm^2^) and a conventional three‐slice sequence with conventional spatial resolution (1.9 × 1.9 mm^2^) were then acquired in 10 patients under rest conditions. Qualitative assessment was performed to assess image quality and perceived signal‐to‐noise ratio (SNR) on a 4‐point scale (0: poor image quality/low SNR; 3: excellent image quality/high SNR), and the number of myocardial segments with diagnostic image quality was recorded. Quantitative measurements of myocardial sharpness and upslope index were performed.

**Results:**

Fat signal leakage was significantly higher for blipped CAIPI than for RF‐CAIPI with GC‐LOLA (7.9% vs. 1.2%, *p* = 0.010). All 10 SMS‐bSSFP perfusion datasets resulted in 16/16 diagnostic myocardial segments. There were no significant differences between the SMS and conventional acquisitions in terms of image quality (2.6 ± 0.6 vs. 2.7 ± 0.2, *p* = 0.8) or perceived SNR (2.8 ± 0.3 vs. 2.7 ± 0.3, *p* = 0.3). Inter‐reader variability was good for both image quality (ICC = 0.84) and perceived SNR (ICC = 0.70). Myocardial sharpness was improved using the SMS sequence compared to the conventional sequence (0.37 ± 0.08 vs 0.32 ± 0.05, *p* < 0.001). There was no significant difference between measurements of upslope index for the SMS and conventional sequences (0.11 ± 0.04 vs. 0.11 ± 0.03, *p* = 0.84).

**Conclusion:**

SMS‐bSSFP with multiband factor 3 and compressed sensing reconstruction enables cardiac MR perfusion imaging with three‐fold increased spatial coverage and improved myocardial sharpness compared to a conventional sequence, without compromising perceived SNR, image quality, upslope index or number of diagnostic segments.

## 1 INTRODUCTION

First‐pass cardiac MR (CMR) perfusion imaging is widely used for the non‐invasive assessment of myocardial ischemia and has demonstrated high accuracy for the diagnosis of hemodynamically significant coronary artery disease when compared against invasive coronary angiography and fractional flow reserve.^1^

A typical myocardial perfusion sequence consists of a dynamic saturation recovery sequence acquired over 50–60 heartbeats.^2^ Contrast is injected at the start of the acquisition, resulting in signal enhancement in regions of the myocardium with normal perfusion. Delayed or reduced myocardial signal enhancement indicates reduced blood supply to the corresponding region(s). The sequence is acquired at stress (during administration of a vasodilatory stress agents such as adenosine) and at rest, to determine the presence and extent of inducible ischemia.

Conventional CMR perfusion acquisitions enable imaging of three to four short axis slices per heartbeat with 2–3 × 2–3 mm^2^ in‐plane resolution, using either a gradient echo, hybrid EPI (hEPI), or balanced SSFP (bSSFP) readout.^2^ The latter may be advantageous at 1.5T due to its high contrast‐to‐noise ratio and SNR efficiency.^3^, ^4^ Enabling full left ventricular (LV) coverage for CMR perfusion is desirable to improve the assessment of myocardial ischemic burden,^5^, ^6^ which has demonstrated important prognostic value.^7^ Previous studies have shown that increased spatial coverage may also improve detection of coronary artery disease^8^ and in particular, detection of apical perfusion defects.^9^ Finally, full LV coverage could improve simultaneous assessment of ischemia and infarction when combined with late gadolinium enhancement imaging.^10^, ^11^ There are also a number of benefits associated with increased in‐plane spatial resolution, such as reduced dark rim artifacts,^12^, ^13^ improved detection of subendocardial perfusion defects,^14^, ^15^ and assessment of transmural perfusion gradients.^15^, ^16^ In addition, we have recently shown that high‐resolution CMR perfusion imaging provides a greater ischemic burden compared to 3D whole‐heart imaging (with lower in‐plane spatial resolution) and, therefore, may be more sensitive for the detection of ischemia.^17^ Thus, there is a desire to achieve full LV coverage and high in plane spatial resolution.

CMR perfusion imaging with full LV coverage has been achieved using 3D imaging with high acceleration.^18^, ^19^, ^20^ However, even with high acceleration factors, the achievable spatial resolution is limited to ∼2.3 × 2.3 mm^2^.^21^ Furthermore, the relatively long acquisition duration (∼200–300 ms^21^) may be more prone to dark rim artifacts and blurring due to cardiac motion.^22^ Simultaneous multi‐slice (SMS) is another technique used for increased spatial coverage, which uses multiband pulses to excite multiple slices simultaneously. Contemporary applications of the technique typically use slice‐dependent RF phase cycling (RF‐CAIPI)^23^ or ”blipped” slice gradients (blipped‐CAIPI)^24^ to effect a shift in the FOV between simultaneously excited slices, thereby maximally exploiting coil sensitivity biases for slice separation.^23^ RF‐CAIPI results in a misaligned frequency response across the simultaneously excited slices which can be corrected for using the gradient‐controlled local Larmor adjustment (GC‐LOLA) technique.^25^ Although GC‐LOLA results in widening of the bSSFP banding artifacts, a number of 1.5T CMR perfusion studies using this technique have not observed any banding artifacts encroaching on the left ventricular myocardium.^26^, ^27^, ^28^ Blipped‐CAIPI has increased theoretical sensitivity to eddy‐current related artifacts due to the additional ”blipped” gradients; however, no visible artifacts were identified with this technique in a prior study.^29^ Another consideration for blipped‐CAIPI is the chemical shift between water and fat, which results in an offset of the slice location of excited fat signal compared to water signal. Since the blipped‐CAIPI gradients are applied in the slice direction, this will give different phases for water and fat spins leading to a split peak in the point spread function of the fat signal relative to the water signal.^30^ The impact of this behavior has not been previously studied for the SMS‐bSSFP blipped CAIPI sequence.

Two previous studies using SMS‐bSSFP for myocardial perfusion imaging at 1.5T have used a multiband factor of 2 (MB2) to enable doubled slice coverage compared to a conventional acquisition. The first study used uniform undersampling with an overall acceleration factor of 5 and in‐plane spatial resolution of 1.9 × 1.9 mm^2^.^26^ The second study implemented a random undersampling scheme and used a compressed sensing reconstruction to reach a higher overall acceleration factor of 11 and a higher in‐plane spatial resolution of 1.4 × 1.4 mm^2^.^27^ In these two studies, the acquisition of only six slices was feasible. However, the long axis of the left ventricle is approximately 90 mm on average,^31^ which would require the acquisition of nine slices to achieve full left ventricular coverage. More recently, SMS‐bSSFP has been extended to a higher multiband factor (MB3) to achieve an all‐systolic perfusion acquisition.^28^ This technique used an acceleration factor of 10 with moderate spatial resolution (2.0 × 2.0 mm^2^); therefore, its potential for full LV coverage with high spatial resolution remains unknown.

In this study, we sought to implement an SMS‐bSSFP perfusion sequence with full LV coverage and high in‐plane spatial resolution at 1.5T. An initial comparison of MB3 acquisitions using RF‐CAIPI with GC‐LOLA and blipped‐CAIPI is performed to assess the behavior of each sequence in the presence of fat. Based on this comparison, the RF‐CAIPI with GC‐LOLA sequence is implemented with a total acceleration factor of 16 and compressed sensing reconstruction. This sequence is acquired at rest in 10 patients to evaluate its potential for myocardial perfusion imaging with full LV coverage (nine slices/heartbeat) and high in‐plane spatial resolution (1.4 × 1.4 mm^2^) at 1.5T.

## 2 METHODS

To achieve full LV coverage while minimizing any associated SNR loss, the SMS‐bSSFP acquisition was extended to MB3, which has previously demonstrated only a marginal increase in g‐factor related noise amplification compared to MB2.^29^ MB3 was implemented for both blipped‐CAIPI and RF‐CAIPI with GC‐LOLA to enable comparison of these two techniques. Blipped‐CAIPI was implemented as in.^29^ Briefly, the slice selective gradient of the bSSFP sequence is modified so that a (+A, +A, −2A) gradient area is added to successive TR periods. The area, A, is a function of the distance of the simultaneously excited slices and it is chosen so that the outer‐most slices are subject to phase shifts of (0, 2π/3, 4π/3) and (0, 4π/3, 2π/3), respectively, relative to the phase imparted to the center slice. Note that the sequence is also balanced, as the re‐phasing gradient, that is, the gradient placed before the next RF pulse, counterbalances the effects of the first. For RF‐CAIPI, three MB3 pulses were calculated, each composed as a superposition of three single band pulses that achieve slice‐specific phase shifts for the outer most slices equal to (0, 2π/3, 4π/3) and (0, 4π/3, 2π/3). The GC‐LOLA correction for MB3 was implemented as previously described.^28^ For both sequences, a three‐fold extended phase FOV was applied so that all three simultaneously excited slices are reconstructed simultaneously on the extended FOV image. Since the simultaneously excited slices 1, 2, and 3 are shifted by 1/3, 0, and −1/3 of the enlarged FOV, respectively, this approach enables slice separation using conventional SENSE/GRAPPA‐like reconstruction approaches.^32^

All experiments were performed on a 1.5T MRI scanner (MAGNETOM Aera, Siemens Healthcare, Erlangen, Germany) using a 32‐element spine array coil and an 18‐element body array coil. The study was approved by the National Research Ethics Service (15/NS/0030) and written informed consent was obtained from all patients.

### 2.1 Comparison of blipped‐CAIPI and RF‐CAIPI in the presence of fat

Blipped‐CAIPI and RF‐CAIPI with GC‐LOLA sequences were acquired in six subjects (four female, two male, mean age 63 ± 5 y) to assess the behavior of these sequences in the presence of fat. Acquisition parameters were equivalent to the perfusion acquisitions described in the following section 2.2, but with a single dynamic frame, one slice group, and no undersampling to avoid any potential slice leakage artifacts related to acceleration. A single‐band RF pulse (rather than a MB pulse) was used to improve visualization of slice leakage artifacts as only one slice (as opposed to three) are excited. For blipped‐CAIPI, the gradients were applied as for a standard MB3 acquisition, and for RF‐CAIPI, the RF phase cycling and GC‐LOLA correction were applied as for a standard MB3 acquisition. Each sequence consisted of a breath‐hold axial acquisition through the abdomen so that the motion of the heart was excluded. The two sequences were acquired with 30 mm inter‐slice distance between simultaneously acquired slices (as required for a nine‐slice perfusion acquisition with full LV coverage and slice thickness of 10 mm), so that there is maximum distance between simultaneously acquired slices. For five patients, the sequences were repeated with 90 mm between simultaneously acquired slices, to illustrate the relationship between the inter‐slice distance and leakage of fat signal.

Quantitative analysis of the fat signal leakage was performed. For each acquired extended FOV image, an region of interest (ROI) was placed in the abdominal subcutaneous fat in the central third of the image (corresponding to the correct location of the excited slice). This ROI was then copied to the top and bottom thirds of the image. The relative ROI signal values in the top third and bottom third of the image were calculated as a percentage of the mean ROI signal value in the central third of the image. The average of these was taken as a measure of the fat signal leakage. Paired t‐tests were used to compare fat signal leakage measurements between blipped‐CAIPI and RF‐CAIPI with GC‐LOLA acquisitions.

### 2.2 RF‐CAIPI SMS‐bSSFP for full LV coverage

**FIGURE 1:**
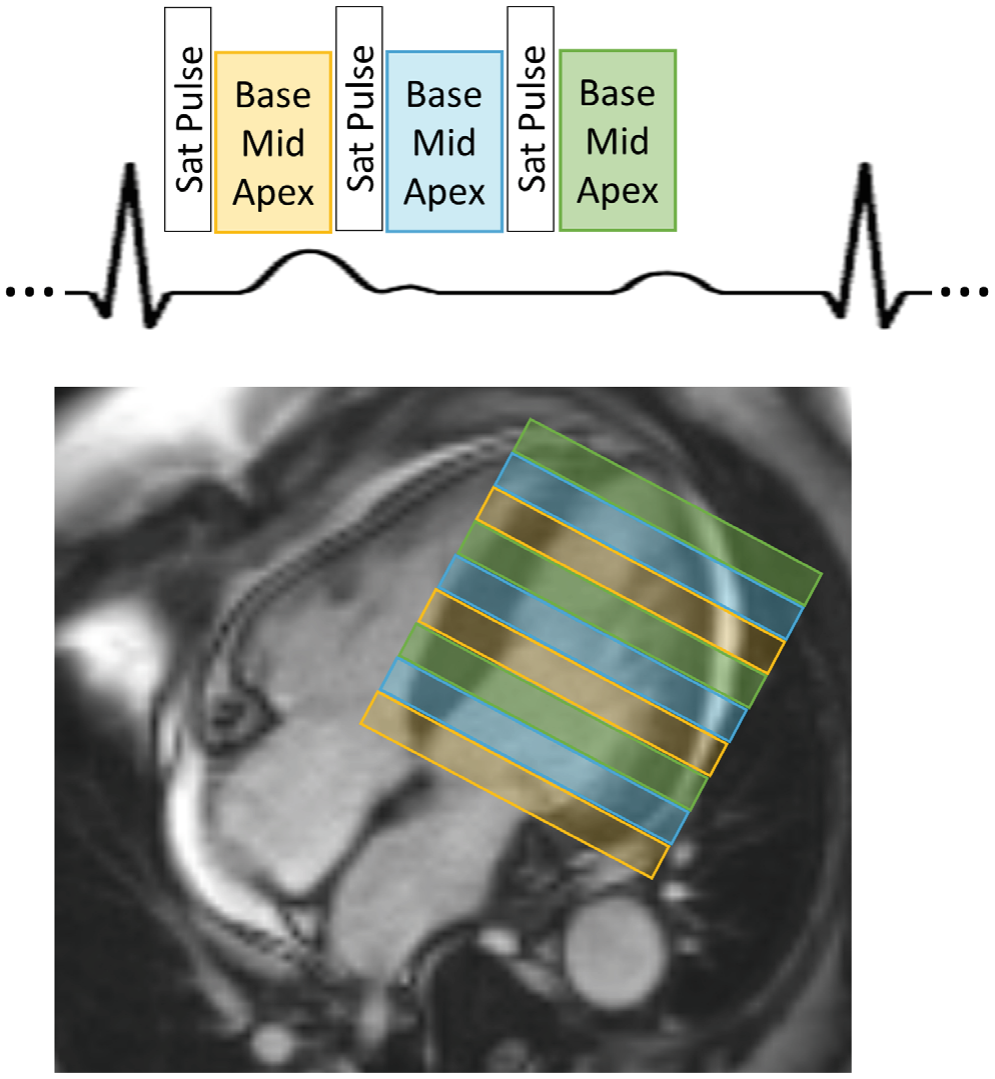
Schematic showing the basic design of the pulse sequence and slice planning for SMS‐bSSFP perfusion imaging with full LV coverage. The acquisition is repeated every heartbeat for 60 dynamic frames

Based on the initial results comparing the two existing SMS‐bSSFP sequences, which demonstrated leakage of fat signal across slices using blipped‐CAIPI, the RF‐CAIPI with GC‐LOLA approach was adopted for the in vivo assessment of SMS‐bSSFP perfusion with full LV coverage. The perfusion sequence was designed with three MB slice groups acquired in each RR interval giving a total of nine slices per heartbeat (Figure 1). For all acquisitions, the slices are acquired in an interleaved fashion, so that there is maximum distance between simultaneously acquired slices. A pseudorandom undersampling algorithm previously developed for SMS‐bSSFP MB2^27^ and extended to MB3^28^ was used to accelerate the sequence. In brief, the central region of k‐space is fully sampled, while the ”peripheral” k‐space lines are pseudo‐randomly under‐sampled to achieve the required acceleration while maintaining the bSSFP steady‐state signal response as well as the k‐space phase ramp requirements for slice‐specific CAIPI shifts. The peripheral k‐space line indices are evenly distributed across three bins, which each correspond to one of the three MB pulses: bin 1 contains 1, 4, 7…; bin 2 contains 2, 5, 8… and bin 3 contains 3, 6, 9… etc. An even number of indices are randomly selected from each of these bins to achieve the required acceleration. The k‐space trajectory is then created by adding the lowest randomly selected k‐space index from each bin sequentially. This process is repeated for each dynamic separately so that incoherence in time is also achieved,^27^ as required for the CS reconstruction framework. Ten fully sampled proton‐density‐weighted gradient‐echo images were acquired prior to the perfusion sequence to provide coil sensitivity information. These maps were acquired in free‐breathing with FOV and spatial resolution matched to the SMS‐bSSFP acquisition. The resultant signal is averaged across the 10 acquisitions to reduce sensitivity to respiratory motion.

The CS reconstruction algorithm^33^ used to reconstruct the undersampled SMS‐bSSFP images consists of a non‐linear iterative algorithm with L1 regularization in spatial and temporal wavelet space:

(1) $$\begin{array}{cc} & {\left\{x_{t}\right\}}_{t=1,\ldots ,T}={\text{argmin}}_{\left\{x_{t}\right\}}\sum_{t=1}^{T}\left({\left\|A_{t}x_{t}-y_{t}\right\|}_{2}^{2}+\lambda_{\omega }{\left\|W_{\omega }x_{t}\right\|}_{1}\right)\\ & \;+\lambda_{\tau }\left\|W_{\tau }{\left\{x_{1}^{T},\ldots ,x_{T}^{T}\right\}}^{T}\right\|1 \end{array}$$

For each time frame, *t*, *x*_*t*_ represents the estimated reconstructed image and *y*_*t*_ represents the measured data. *A*_*t*_ is the encoding operator at time frame *t*, which includes the coil sensitivity profiles, Fourier transform and the pseudorandom undersampling mask. *W*_*ω*_ and *W*_*τ*_ represent the spatial and temporal wavelet transformations, and *λ*_*ω*_ and *λ*_*τ*_ represent the spatial and temporal regularization parameters. *λ*_*ω*_, *λ*_*τ*_ and the number of fully sampled k‐space center lines were empirically optimized to 0.0001, 0.005, and 18 respectively.

Twelve patients referred for a clinical contrast‐enhanced non‐stress CMR examination were prospectively recruited for the study, of which 10 patients (6 female, 4 male, mean age 44.7 ± 16.8 y) were included. Two patients were excluded from the analysis due to non‐compliance with the breath‐hold instruction (see below). The 10 patients included in the study were referred for evaluation of myocardial viability (*N* = 1), myocarditis (*N* = 5), arrhythmogenic right ventricular cardiomyopathy (*N* = 2), suspected thrombus (*N* = 1), and intracardiac shunt (*N* = 1). Two perfusion sequences (a conventional three‐slice sequence and the proposed SMS‐bSSFP nine‐slice sequence) were acquired at rest in a random order, ensuring a minimum delay of 10 min between the two acquisitions. A 0.075 mmol/kg dose of a gadolinium‐based contrast agent (Gadovist, Bayer, Berlin, Germany) was injected during each perfusion sequence, and patients were instructed to hold their breath for the duration of the first pass of the contrast agent (approximately 20 frames).

The three‐slice acquisition was planned on the systolic phase of two‐, three‐, and four‐chamber cine images, ensuring inclusion of a basal, mid, and apical slice. The nine‐slice sequence was planned on the end‐diastolic phase of the two‐, three‐, and four‐chamber cine images, ensuring maximum coverage of the left ventricle (LV). The slice acquisition order was prescribed to achieve the maximum distance between simultaneously acquired slices for all slice groups (Figure 1). The proposed sequence had higher in‐plane spatial resolution compared with the conventional three‐slice sequence (1.4 × 1.4 mm^2^ vs. 1.9 × 1.9 mm^2^), a higher number of slices (nine slices vs. three slices) and a higher total acceleration factor (16 vs. 3). The conventional sequence used standard in‐plane generalized autocalibrating partial parallel acquisition (GRAPPA) acceleration.^34^ For the SMS acquisition, the total acceleration factor is the product of the slice acceleration (or MB) factor^3^ and the effective in‐plane acceleration factor (5.33). All other parameters were largely consistent for the two sequences: FOV = 360 × 360 mm^2^; TR/TE/flip angle = 2.50 ms/1.04 ms/40° (conventional) and 2.95 ms/1.26 ms/40° (SMS); saturation time = 99 ms; slice thickness = 10 mm; slice gap = 5–20 mm (conventional) and 0 mm (SMS); readout duration per slice/slice group = 156 ms (conventional) and 141 ms (SMS); acquisition duration for all slices = 564 ms (conventional) and 540 ms (SMS); and bandwidth = 1302 Hz/Px. For each acquisition, 60 dynamic frames were acquired. Data acquired with the conventional sequence were reconstructed using standard GRAPPA reconstruction,^34^ while the SMS data were reconstructed using the compressed sensing reconstruction algorithm outlined in the previous section. All datasets were assessed qualitatively by three readers (A.C., M.S.N., and A.D.V.) with 15, 5, and 7 y of CMR experience, respectively. All readers were blinded to the patient information and datasets were loaded in a randomized order. Image quality was assessed over the duration of the first‐pass for each myocardial slice on a 4‐point scale: 0 = poor image quality/non‐diagnostic; 1 = major artifacts present but not limiting diagnosis; 2 = minor artifacts present but not limiting diagnosis; 3 = excellent image quality. Slices acquired outside the LV were not assessed. The percentage of slices with each image quality score was calculated. Each reader also recorded an overall perceived SNR score for each SMS and conventional dataset on a 4‐point scale: 0: very poor SNR, non‐diagnostic image quality; 1: major noise present but not limiting diagnosis; 2: minor noise present but not limiting diagnosis; 3: high SNR. The percentage of patients with each perceived SNR score was calculated. Finally, a binary score of diagnostic or non‐diagnostic image quality was recorded for each of the 16 American Heart Association (AHA) myocardial segments.^35^

Quantitative measurements of sharpness were performed as described in previous studies^27^, ^36^ to assess whether the nominally higher resolution of the SMS sequence resulted in improved sharpness of the border between the blood pool and the myocardium. Briefly, the sharpness index is the reciprocal of the distance over which the signal intensity profile drawn across a high‐contrast interface drops from 80% to 20% of the signal range. Tightly spaced signal profiles were drawn across the blood‐septal interface at the dynamic corresponding to peak LV blood pool enhancement and the average sharpness index measured across all profiles was recorded. Measurements were performed on all three slices of the conventional acquisition and on the three SMS slices that were most closely matched in terms of cardiac phase and slice location, to avoid any potential bias due to cardiac motion. The upslope index was measured on a mid‐ventricular slice as a surrogate assessment of temporal fidelity of the contrast enhancement. The upslope of the signal intensity‐time curve is calculated as the maximum positive gradient of the curve over four consecutive time‐points. The upslope index is then calculated as the ratio of the upslope measured from a myocardial region of interest (ROI) and left ventricular blood pool ROI.^37^ Late gadolinium enhancement (LGE) acquisitions were acquired as part of the standard clinical protocol. For any patients where scar was present, LGE was compared qualitatively against any defects observed in perfusion acquisitions.

Statistical analysis was performed using SPSS (SPSS Version 25, IBM, USA). The Wilcoxon signed ranks test was used to compare image quality and perceived SNR scores. Inter‐reader agreement of these subjective measures was assessed using the two‐way mixed average measuresintraclass correlation coefficient (ICC). The paired *t*‐test was used to compare measurements of the sharpness index and upslope index. Two‐tailed values of *p* < 0.05 were considered significant for all statistical tests.

## 3 RESULTS

### 3.1 Comparison of blipped‐CAIPI and RF‐CAIPI with GC‐LOLA in the presence of fat

**FIGURE 2:**
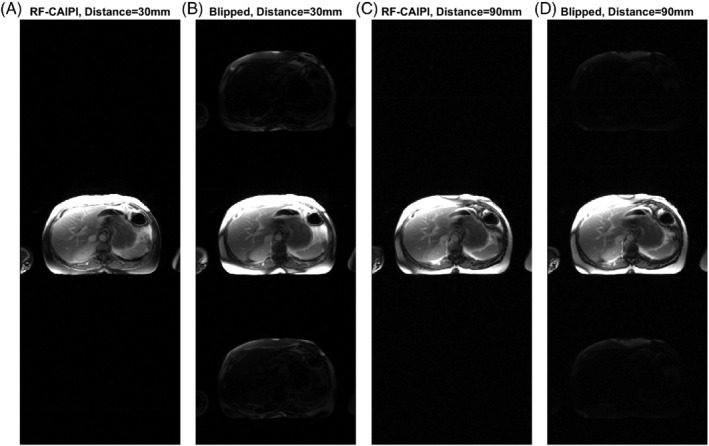
RF‐CAIPI with GC‐LOLA (A,C) and blipped‐CAIPI (B,D) images acquired with a single band pulse (with MB3 blipped gradients for the blipped‐CAIPI acquisition and MB3 CAIPI phases and GC‐LOLA correction for the RF‐CAIPI acquisition). A,B, Acquired as for an SMS3 myocardial perfusion acquisition, with 30 mm inter‐slice distance between the three prescribed slices in the SMS slice group. C,D, Acquired with an inter‐slice distance of 90 mm. For the blipped CAIPI acquisition, there is significant leakage of the fat signal from the acquired slice into the top and bottom thirds of the image (corresponding to the locations of the other two simultaneously acquired slices of a MB3 acquisition) when an inter‐slice distance of 30 mm is applied. This is reduced when the inter‐slice distance is increased to 90 mm, which uses smaller blipped gradients

Figure 2 shows a comparison of the RF‐CAIPI with GC‐LOLA acquisitions (Figure 2A,C) and blipped‐CAIPI acquisitions (Figure 2B,D). For all acquisitions, the single‐band images are shown reconstructed on a three‐fold extended FOV image to demonstrate any signal leakage that would occur across slices in a MB3 acquisition. Leakage of the fat signal is observed in the top and bottom thirds of both blipped‐CAIPI acquisitions, corresponding to the locations of the two other simultaneously acquired slices in a MB3 acquisition. Across all patients, the fat signal leakage was significantly higher for blipped CAIPI than for RF‐CAIPI with GC‐LOLA for the 30 mm inter‐slice distance acquisition (7.9% vs. 1.2%, *p* = 0.010) and for the 90 mm inter‐slice distance acquisition (3.2% vs. 1.2%, *p* = 0.003). The fat signal leakage was significantly higher for the blipped CAIPI acquisition with 30 mm inter‐slice distance than with 90 mm inter‐slice distance (8.4% vs. 3.2%, *p* = 0.049).

### 3.2 RF‐CAIPI SMS‐bSSFP for full LV coverage

**FIGURE 3:**
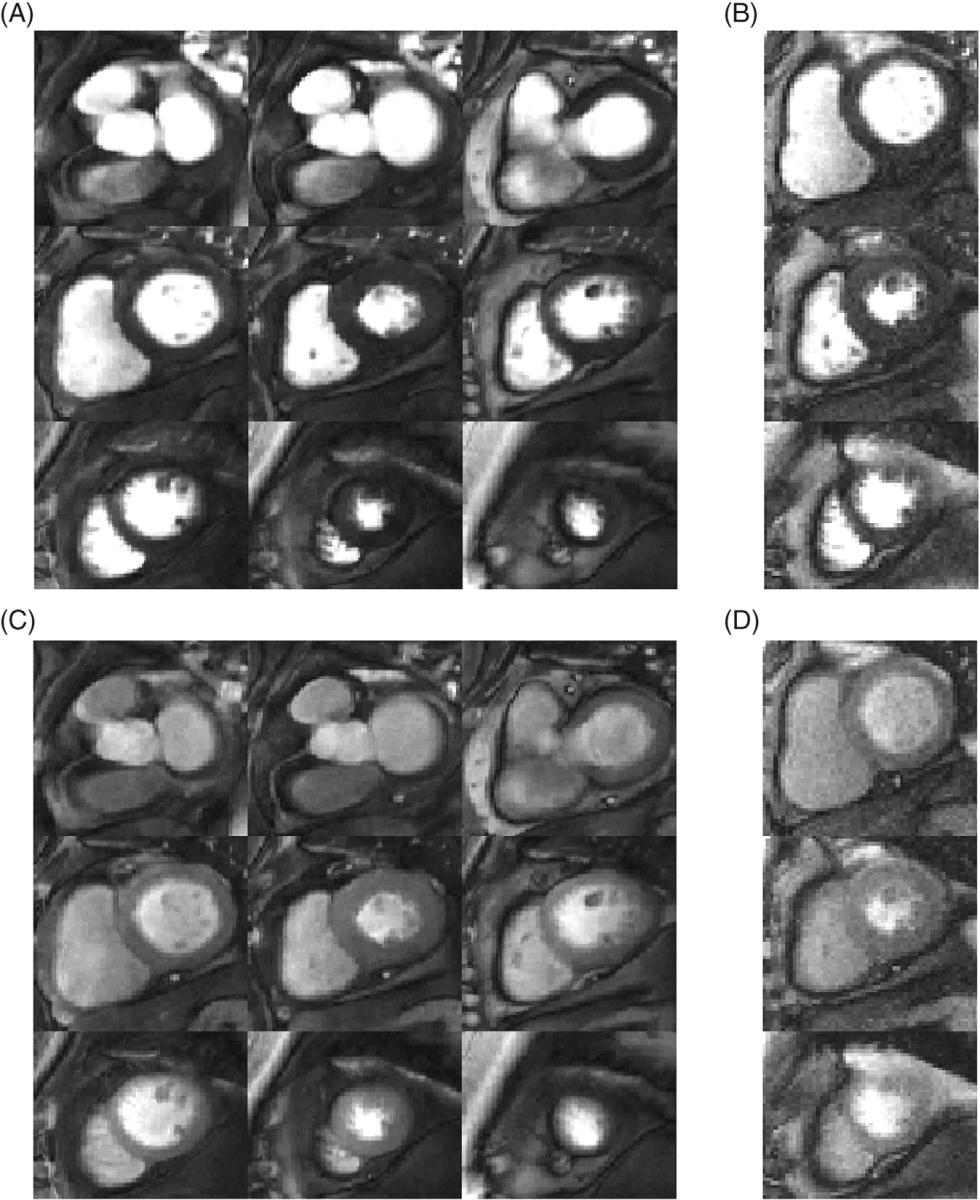
Example perfusion images acquired in a patient under rest conditions, shown at peak enhancement of the left ventricular blood pool (A,B) and at peak myocardial enhancement (C,D). The nine‐slice high resolution (1.4 × 1.4 mm^2^) SMS sequence (A,C) was acquired first and the three‐slice conventional sequence with in‐plane resolution = 1.9 × 1.9 mm^2^ and GRAPPA reconstruction (B,D) was acquired after a 10‐min delay. For each dataset, the window width and level were kept constant for both timepoints. Image quality of all myocardial slices and both acquisitions were scored as 3.0 (= excellent image quality/no artifacts), except for the apical slice of the three‐slice acquisition, which was scored as 2.0 (= minor artifacts and of diagnostic quality), due to the partially obscured lateral wall in this slice

**FIGURE 4:**
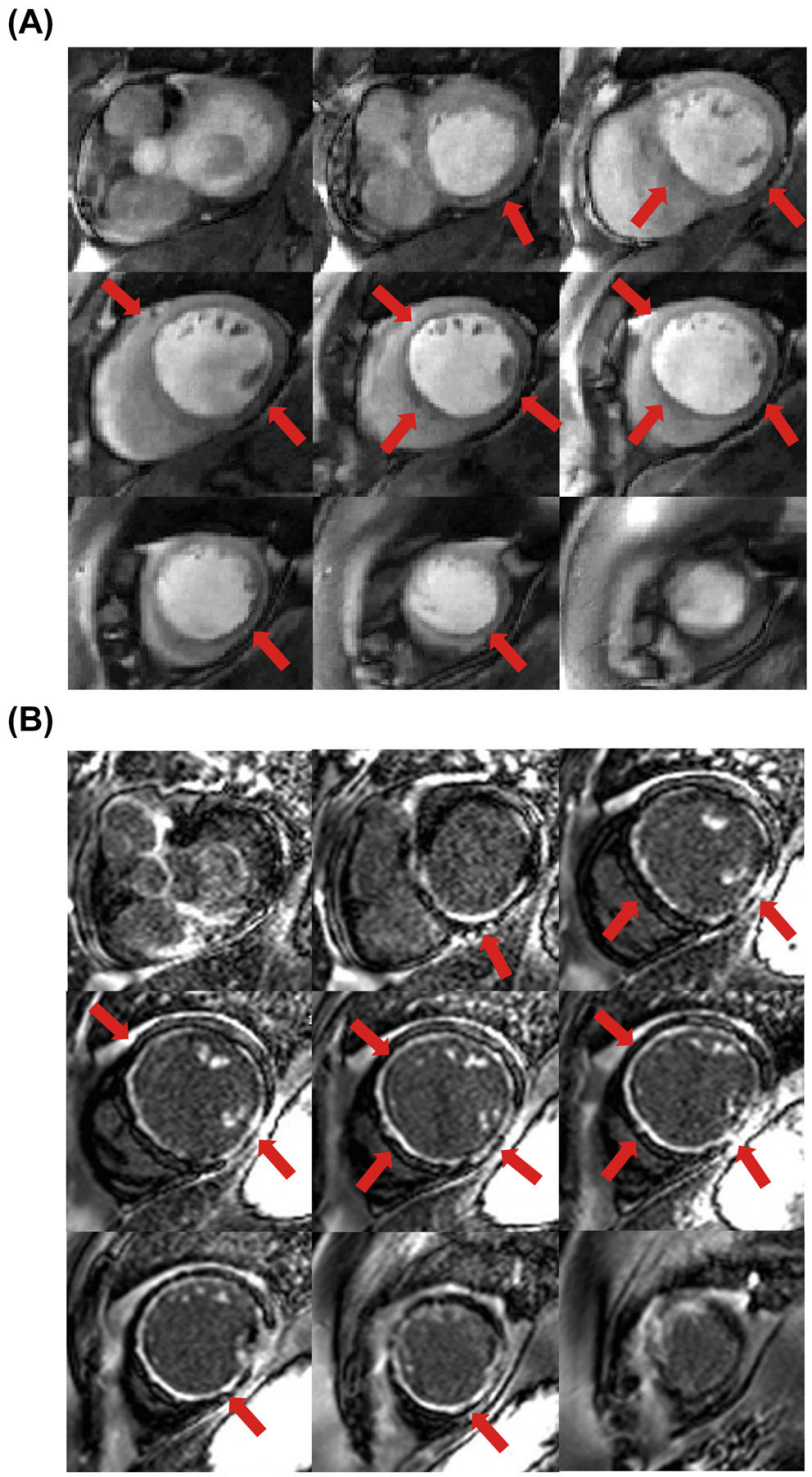
A, SMS‐bSSFP perfusion images acquired under rest conditions in a patient with extensive subendocardial perfusion defects. B, Dark‐blood late gadolinium enhancement images^49^ acquired in the same patient. Slice coverage is well‐matched between the two acquisitions. Regions of reduced myocardial signal enhancement indicated by red arrows on the perfusion images (A) match bright regions indicating scar on late gadolinium enhancement images (B)

**FIGURE 5:**
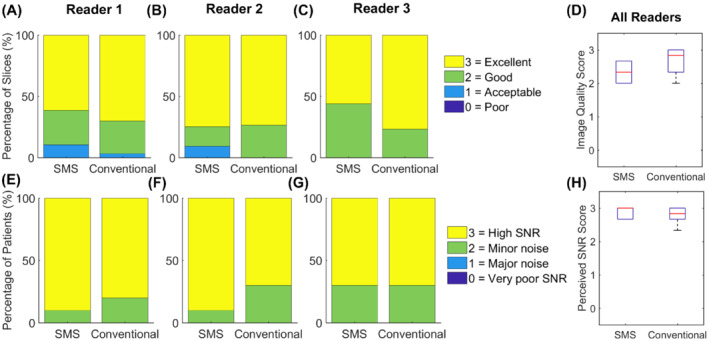
Results from qualitative assessment in 10 patients, scored by three expert readers. A–C, Distribution of image quality scores shown as a percentage of total number of slices over all patients. E–G, Distribution of perceived SNR scores shown as a percentage of total number of patients. D,H, Boxplots showing the distribution of average image quality scores (D) and perceived SNR scores (H) for 10 patients as assessed by three expert readers. There was no significant difference between the nine‐slice SMS sequence and three‐slice conventional sequence in terms of average image quality scores (*p* = 0.8) or average perceived SNR scores (*p* = 0.3)

**FIGURE 6:**
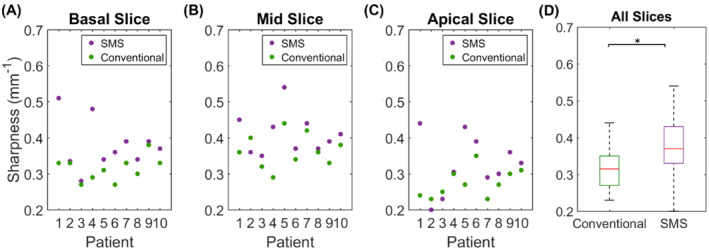
A–C, Myocardial sharpness measurements for each slice and each patient using the three‐slice conventional sequence (green dots) and the nine‐slice SMS sequence (purple dots). D, Boxplots showing distribution of sharpness measurements across all slices and all patients. Sharpness was significantly improved across all slices using the proposed high‐resolution SMS sequence

Both the proposed SMS and conventional perfusion sequences were acquired in all patients. The two patients who were excluded from the analysis were breathing throughout the first pass of the contrast agent. Images from these patients are shown in Supporting Information Videos S1 and S2, which are available online. Example images acquired in a patient using the proposed SMS sequence and the conventional sequence at peak LV blood pool and peak myocardial signal enhancement are shown in Figure 3. For this case, the SMS sequence (Figure 3A and C) was acquired first. Figure 3B and D show corresponding conventional perfusion images acquired in the same patient. For both acquisitions and all readers for this case, the image score across all myocardial slices was 3.0 (= excellent image quality/no artifacts), except for the apical slice of the three‐slice acquisition, which was scored as 2.0 (=minor artifacts and of diagnostic quality), due to the partially obscured lateral wall in this slice. Videos showing the entire first‐pass for each sequence are included as Supporting Information (Supporting Information Video S3). Images from another patient, where the conventional sequence was acquired first, are shown in Supporting Information Figure S1 (and Supporting Information Video S4). For this patient, nine contiguous 10 mm slices were required for full LV coverage, which was achieved for the SMS sequence using a MB factor of 3. The average image quality scores across all myocardial slices for the SMS acquisition were 3.0, 3.0, and 2.4 for readers 1, 2, and 3, respectively. For the conventional acquisition, the corresponding scores were 3.0, 3.0, and 3.0. One patient had extensive subendocardial scar (Figure 4). For this patient, all readers scored all slices of the SMS acquisition as excellent image quality (3.0). The slices are well‐matched between the LGE and SMS perfusion acquisitions and hyperintense regions indicating scar on LGE images are matched by regions of low signal enhancement on SMS perfusion images. No other subjects included in the study presented with myocardial LGE. Across all patients, all AHA segments were reported as diagnostic for both the SMS and conventional acquisitions. Bar charts showing the distribution of qualitative assessment scores for each reader are shown in Figure 5A–C and E–G. These show the percentage of slices with each image quality score and the percentage of patients with each perceived SNR score. Inter‐reader variability was good for both image quality (ICC = 0.84, 95% confidence interval = [0.66–0.93]) and perceived SNR (ICC = 0.70, 95% confidence interval = [0.36–0.87]). The distribution of image quality and perceived SNR scores averaged across the three readers are presented as boxplots in Figure 5D and H, respectively. There were no significant differences observed between the SMS and conventional acquisitions in terms of image quality (2.6 ± 0.6 vs. 2.7 ± 0.2, *p* = 0.8) or perceived SNR (2.8 ± 0.3 vs. 2.7 ± 0.3, *p* = 0.3). Sharpness measurements are plotted in Figure 6A–C) for each slice, patient, and sequence. Across all slices and all patients, measurements of sharpness were higher with the nine‐slice sequence than with the three‐slice sequence (0.37 ± 0.08 vs 0.32 ± 0.05, *p* < 0.001, Figure 6D). There was no significant difference between measurements of upslope index for the SMS and conventional sequences (0.11 ± 0.04 vs. 0.11 ± 0.03,*p* = 0.84).

## 4 DISCUSSION

In this study, we implemented and evaluated a high‐resolution SMS‐bSSFP perfusion sequence with full LV coverage at 1.5T. Image quality, perceived SNR, and number of diagnostic segments were not significantly different to conventional three‐slice perfusion acquisition, while the proposed sequence demonstrated improved sharpness and a three‐fold increase in spatial coverage.

The comparison between blipped‐CAIPI and RF‐CAIPI with GC‐LOLA in this study demonstrated that there is significant leakage of fat signal using a blipped‐CAIPI SMS‐bSSFP acquisition in the body, while no leakage was observed for the RF‐CAIPI acquisition. Using a larger distance between slices results in a significant decrease in the slice leakage, as the blipped‐CAIPI gradient moment is inversely proportional to slice distance. The chemical shift between fat and water is linearly related to field strength; thus, the phase offset accumulated as a result of the blipped gradients and the resultant leakage of fat signal across slices will also be linearly related to field strength. We would like to note that while a similar effect is present for blipped‐CAIPI EPI sequences, which are commonly used for acquisitions in the brain, there is limited fat in this region, and the fat signal is less significant than for typical sequences used in cardiac perfusion. Therefore, this is not likely to be a major limitation for blipped‐CAIPI in the brain.

Several previous studies have presented CMR perfusion with full LV coverage using 3D acquisitions.^5^, ^6^, ^21^ One benefit of the proposed 2D SMS technique over these 3D techniques is the ability to simultaneously achieve high in‐plane spatial resolution, which can improve the detection of subendocardial defects and reduce the presence of dark rim artifacts,^13^ and full LV coverage. Other 2D SMS techniques have been proposed for full LV coverage CMR perfusion, using radial or spiral sampling in combination with a gradient echo readout.^38^, ^39^, ^40^ At 3T, an in‐plane spatial resolution of 1.25 × 1.25 mm^2^ has been achieved.^39^ However, at 1.5T, the in‐plane spatial resolution of these techniques has been limited to ∼2.0 × 2.0 mm^2^. To achieve a higher spatial resolution in the current study (1.4 × 1.4 mm^2^), a high total acceleration factor of 16 was used. The ability to achieve such a high acceleration factor can be partly attributed to the bSSFP readout used, which has a higher intrinsic SNR compared to gradient echo.^3^, ^4^ A potential disadvantage of bSSFP compared to gradient echo is the presence of dark band artifacts in regions of off‐resonance. However, these artifacts can be largely avoided at 1.5T by using high order shimming and careful placement of the shim box over the LV. We followed this strategy in the current study and did not observe any dark bands obscuring the LV myocardium.

Implementing higher multiband factors could enable acquisition with an increased number of slices with comparable in‐plane resolution and reduced slice thickness, which would reduce the significance of partial volume effects to a certain extent. However, this would result in decreased intrinsic SNR due to several factors: smaller acquired voxel size, further increased g‐factor amplification and a limit on the maximum achievable flip angle due to limits on the peak B1. Since we wished to maintain comparable image quality and high perceived SNR, we limited the scope of the study to MB3, for which only a relatively minor increase in g‐factor penalty is expected.^29^

Planning of the slice locations for a conventional three‐slice perfusion sequence is non‐trivial, since the three slices are acquired at different phases of the cardiac cycle. This can lead to a discrepancy between prescribed and acquired slice locations and may result in the acquisition of two mid‐ventricular slices and a missing basal or apical slice. The planning of the SMS nine‐slice sequence is more straightforward in this regard, as the nine contiguous slices are centered on the end‐diastolic phase of two‐, three‐, and four‐chamber cine images, ensuring full LV coverage of the heart at the point in the cardiac cycle of maximum diastolic volume. It is acknowledged that in practice there may be some degree of overlap and/or gaps between the acquired slices due to through‐plane motion between cardiac phases. The magnitude of this effect and the impact on assessment of total ischemic burden will need to be evaluated in future studies.

The perfusion acquisition with full LV coverage shown in Supporting Information Figure S1 demonstrates the potential of this sequence to allow for a direct comparison with late gadolinium enhancement imaging. This could help to distinguish between regions of inducible perfusion defects and scar, which could also potentially diminish the requirement for a rest perfusion acquisition.

One of the major remaining limitations of the proposed sequence is the sensitivity to respiratory motion. The strong temporal regularization which is applied in the reconstruction process results in temporal blurring in the presence of motion between dynamic frames. A reconstruction framework integrating motion compensation could reduce the sensitivity to breathing motion and could enable free‐breathing acquisitions, which would be particularly beneficial for patients who cannot sustain a breath‐hold during the first pass.^41^, ^42^ Such retrospective motion compensation strategies cannot, however, correct for through‐plane motion. One method to reduce through‐plane motion is prospective slice tracking using a diaphragmatic navigator,^43^, ^44^ which could prospectively correct for both through‐ and in‐plane motion of the heart due to respiratory motion.

A three‐fold extended FOV was used for SMS acquisition in this study. This renders the reconstruction similar to an in‐plane acceleration problem, which can simplify integration with other inline post‐processing steps, such as motion correction.^26^ However, this framework prevents the integration of phase modulation in the data fidelity term, as all slices (with their respective phase modulations) are reconstructed as a single image.

The reconstruction time of the SMS nine‐slice sequence is currently very long (∼1.5 h per dataset). This could limit the translation to clinical practice when inline reconstruction on the scanner is required. It is noted that a graphics processing unit (GPU) implementation of the reconstruction framework could significantly reduce the reconstruction time in the future.

The use of temporal regularization will inevitably introduce a degree of smoothing across dynamics, which may affect the temporal fidelity of the reconstruction. Since it was not possible to obtain a reference for the true signal during the SMS acquisition, the upslope index was measured to assess the temporal fidelity of the contrast enhancement. While it is acknowledged that this is only a surrogate measurement of temporal fidelity, it is a relevant parameter that has been used extensively for semi‐quantitative assessment of myocardial perfusion imaging in previous studies.^37^, ^45^, ^46^

While not assessed in this study, quantification of myocardial blood flow (MBF) from CMR perfusion imaging with full LV coverage could offer an alternative to MBF quantification from positron emission tomography, with higher in‐plane spatial resolution. Furthermore, CMR perfusion does not expose the patient to ionizing radiation, and follow‐up measurements of MBF could be used to assess and compare the effectiveness of different interventions for treatment of coronary artery disease. Accurate quantification of MBF from CMR perfusion imaging requires a linear relationship between signal intensity and gadolinium concentration for the blood pool and myocardium, which can be achieved using dual‐bolus^47^ or dual‐sequence^48^ techniques, both of which would be compatible with the proposed SMS nine‐slice sequence in this study.

Our study has some limitations. A relatively small number of patients without suspected coronary artery disease were included in this study. In addition, all acquisitions were performed under rest conditions. In one patient, fixed perfusion defects correlated with scar on late gadolinium enhanced images, however evaluation of the technique under stress conditions and in patients with suspected coronary artery disease is warranted to evaluate the diagnostic confidence in the case of more subtle defects. It is noted that the acquisition duration (546 ms) is short enough for heart rates up to 110 beats per minute, which is likely acceptable within clinical routine. Acquisitions at higher heart rates than 110 beats per minute may require a small reduction in spatial resolution.

## 5 CONCLUSIONS

SMS‐bSSFP with MB factor 3 and compressed sensing reconstruction enables CMR perfusion imaging with high spatial resolution and full LV coverage at 1.5T. The proposed sequence provided three‐fold increased spatial coverageand improved myocardial sharpness without compromising perceived SNR, image quality, or number of diagnostic segments compared to a conventional perfusion sequence.

## CONFLICT OF INTEREST

Peter Speier, Radhouene Neji, Karl P Kunze and Daniel Stäb are employees of Siemens Healthineers.

## Supporting information


**Figure S1.** Rest perfusion images acquired in a patient shown at peak enhancement of the left ventricular blood pool (a‐b) and at peak myocardial enhancement (c‐d). The 3‐slice conventional sequence with in‐plane resolution = 2.0 × 2.0 mm^2^ and GRAPPA reconstruction (b, d) was acquired first and the 9‐slice high resolution (1.4 × 1.4 mm^2^) SMS sequence (a, c) was acquired after a 10‐min delay. For this case, 9 slices were required for full LV coverage, which was achieved for SMS sequence witha MB factor of 3. Comparable image quality was achieved for both acquisitions.

**Video S1** Video of perfusion images acquired in a patient who was gradually exhaling during the first‐pass.

**Video S2** Video of perfusion images acquired in a patient who was breathing throughout the first‐pass acquisition.

**Video S3** Video of perfusion images acquired in the patient presented in Figure 3.

**Video S4** Video of perfusion images acquired in the patient presented in Supporting Information Figure S1.

## Data Availability

The data that support the findings of this study are available from the corresponding author (S.R.) upon reasonable request.
