# Supplementary material for: Simultaneous multislice steady‐state free precession myocardial perfusion with full left ventricular coverage and high resolution at 1.5 T
Source: Magn Reson Med. 2022 Mar 28;88(2):663–75. doi: 10.1002/mrm.29229 (PMC9310832; doi:10.1002/mrm.29229)
Supplement: Supplementary file 1 — Figure S1. Rest perfusion images acquired in a patient shown at peak enhancement of the left ventricular blood pool (a‐b) and at peak myocardial enhancement (c‐d). The 3‐slice conventional sequence with in‐plane resolution = 2.0 × 2.0 mm2 and GRAPPA reconstruction (b, d) was acquired first and the 9‐slice high resolution (1.4 × 1.4 mm2) SMS sequence (a, c) was acquired after a 10‐min delay. For this case, 9 slices were required for full LV coverage, which was achieved for SMS sequence witha MB factor of 3. Comparable image quality was achieved for both acquisitions. [file MRM-88-663-s001.docx]

**SUPPORTING INFORMATION**


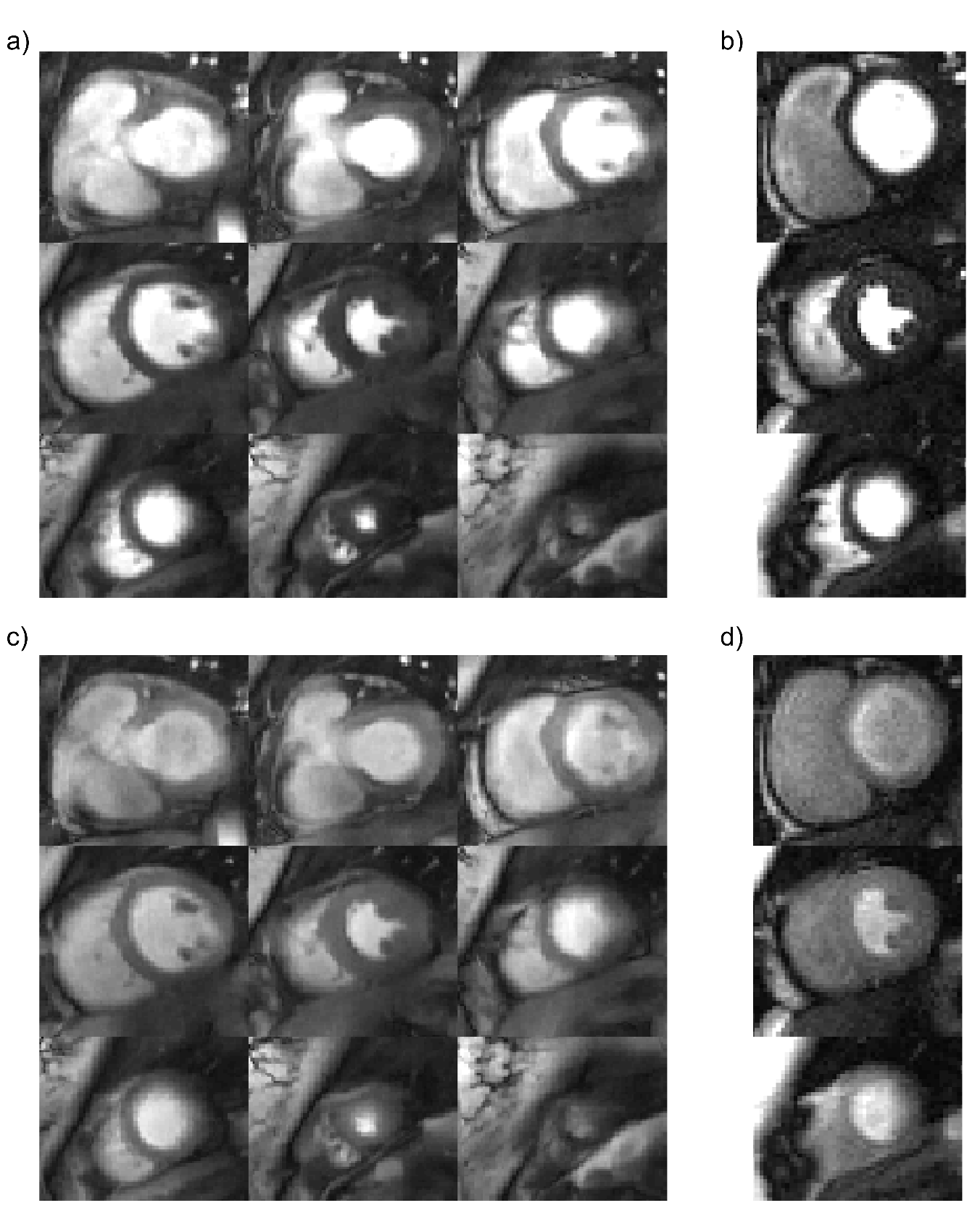
Supporting Information Figure S1: Rest perfusion images acquired in a patient shown at peak enhancement of the left ventricular blood pool (a-b) and at peak myocardial enhancement (c-d). The 3-slice conventional sequence with in-plane resolution = 1.9 x 1.9 mm^2^ and GRAPPA reconstruction (b, d) was acquired first and the 9-slice high resolution (1.4 x 1.4 mm^2^) SMS sequence (a, c) was acquired after a 10-minute delay. For this case, 9 slices were required for whole-heart coverage, which was achieved for SMS sequence with a MB factor of 3. Comparable image quality was achieved for both acquisitions.
