# Supplementary figures and images for: Simultaneous multislice steady‐state free precession myocardial perfusion with full left ventricular coverage and high resolution at 1.5 T
Source: Magn Reson Med. 2022 Mar 28;88(2):663–75. doi: 10.1002/mrm.29229 (PMC9310832; doi:10.1002/mrm.29229)

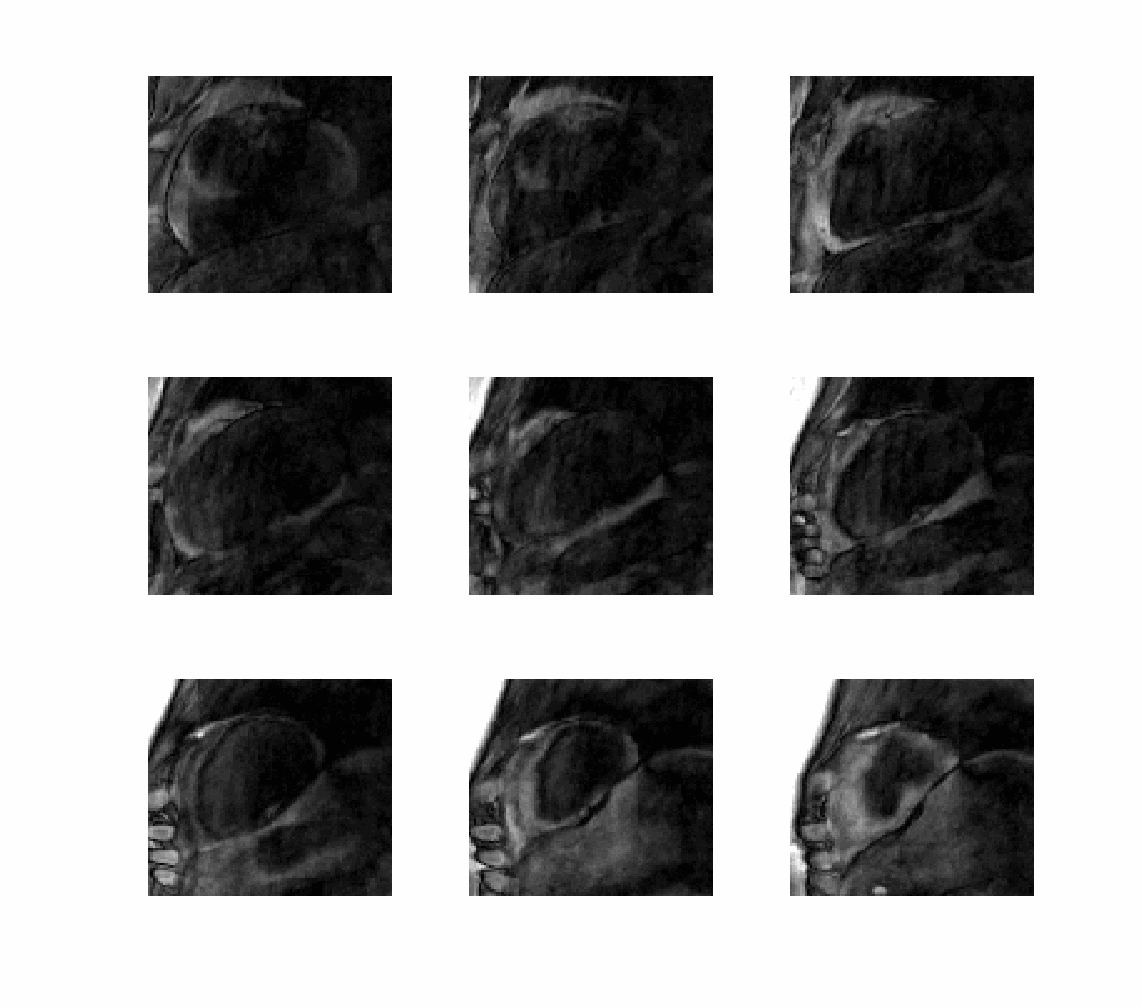

Supplement: Supplementary file 2 — Video S1 Video of perfusion images acquired in a patient who was gradually exhaling during the first‐pass. [file MRM-88-663-s002.gif]

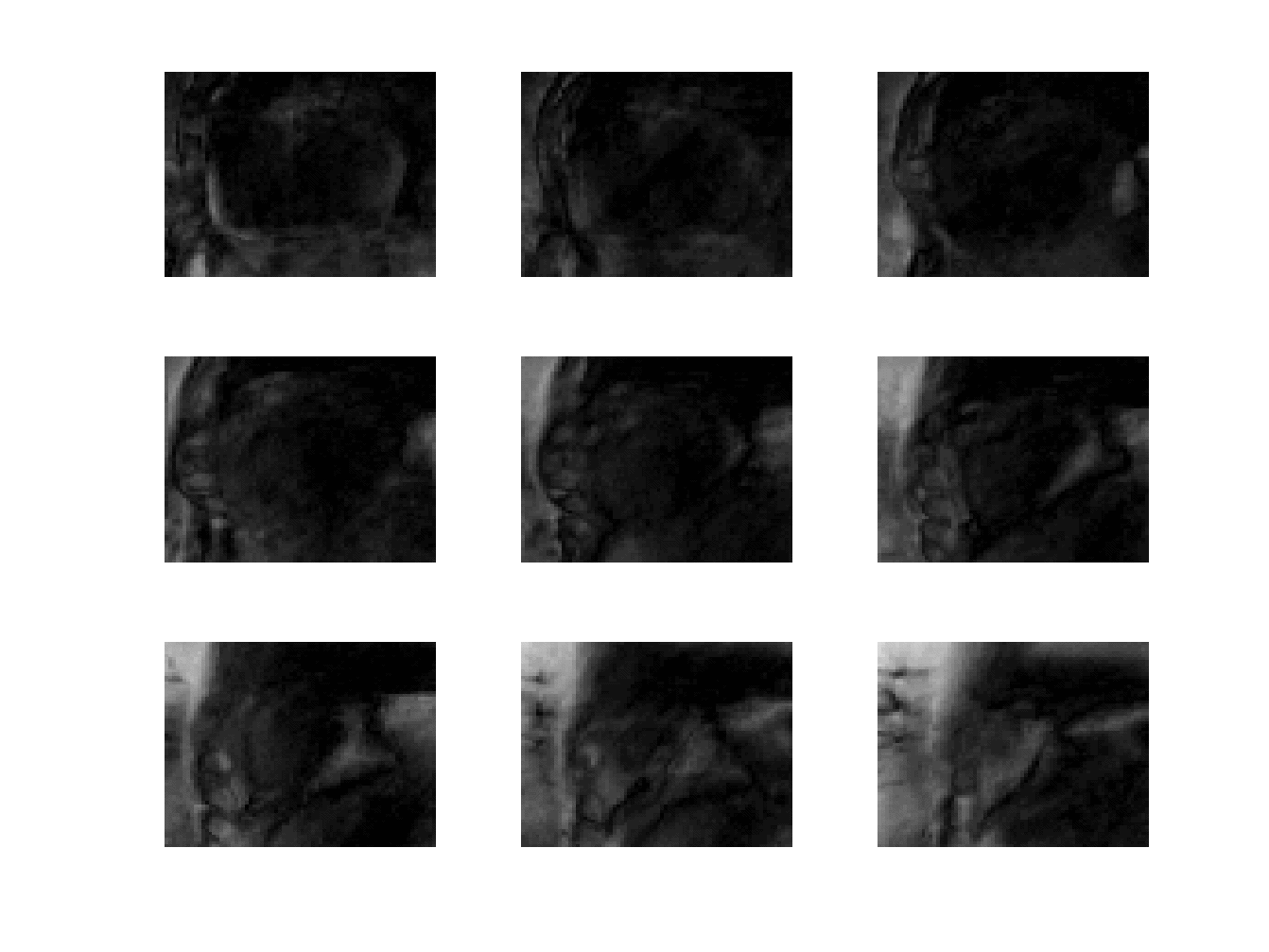

Supplement: Supplementary file 3 — Video S2 Video of perfusion images acquired in a patient who was breathing throughout the first‐pass acquisition. [file MRM-88-663-s005.gif]

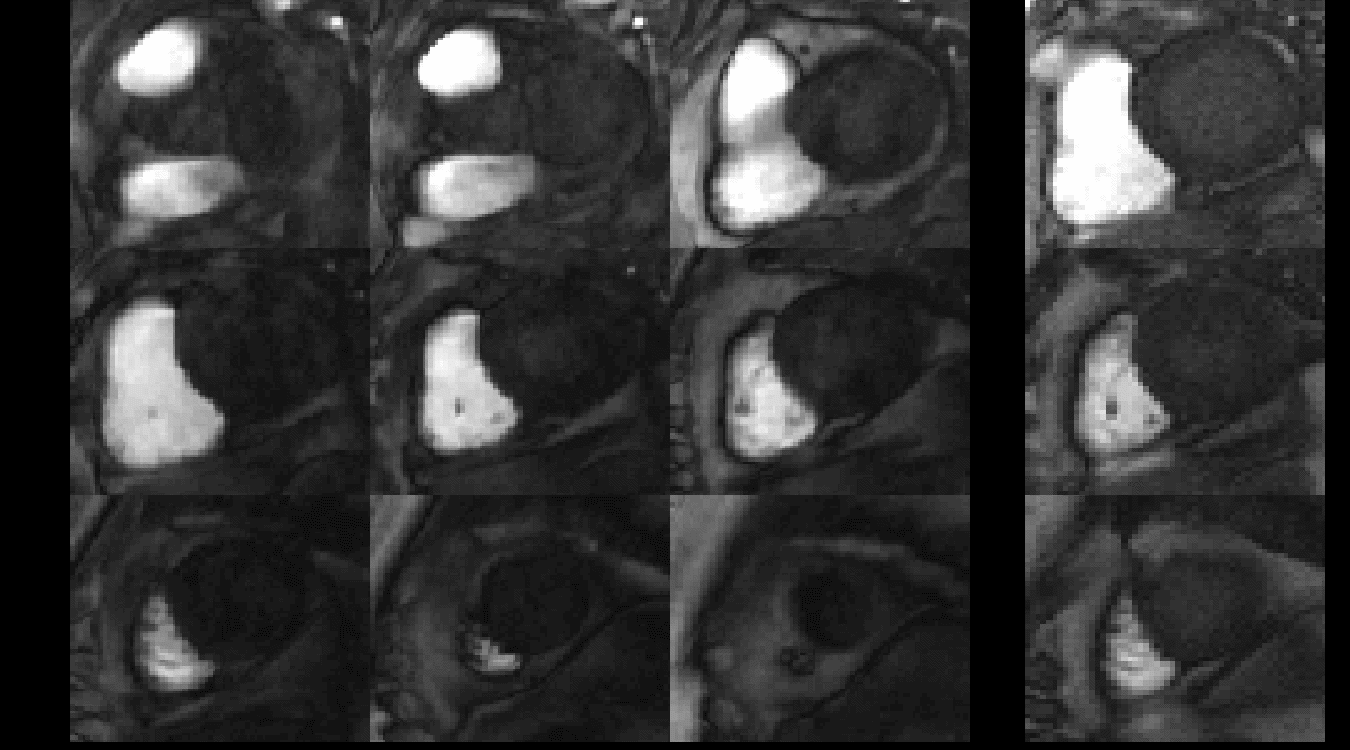

Supplement: Supplementary file 4 — Video S3 Video of perfusion images acquired in the patient presented in Figure 3. [file MRM-88-663-s003.gif]

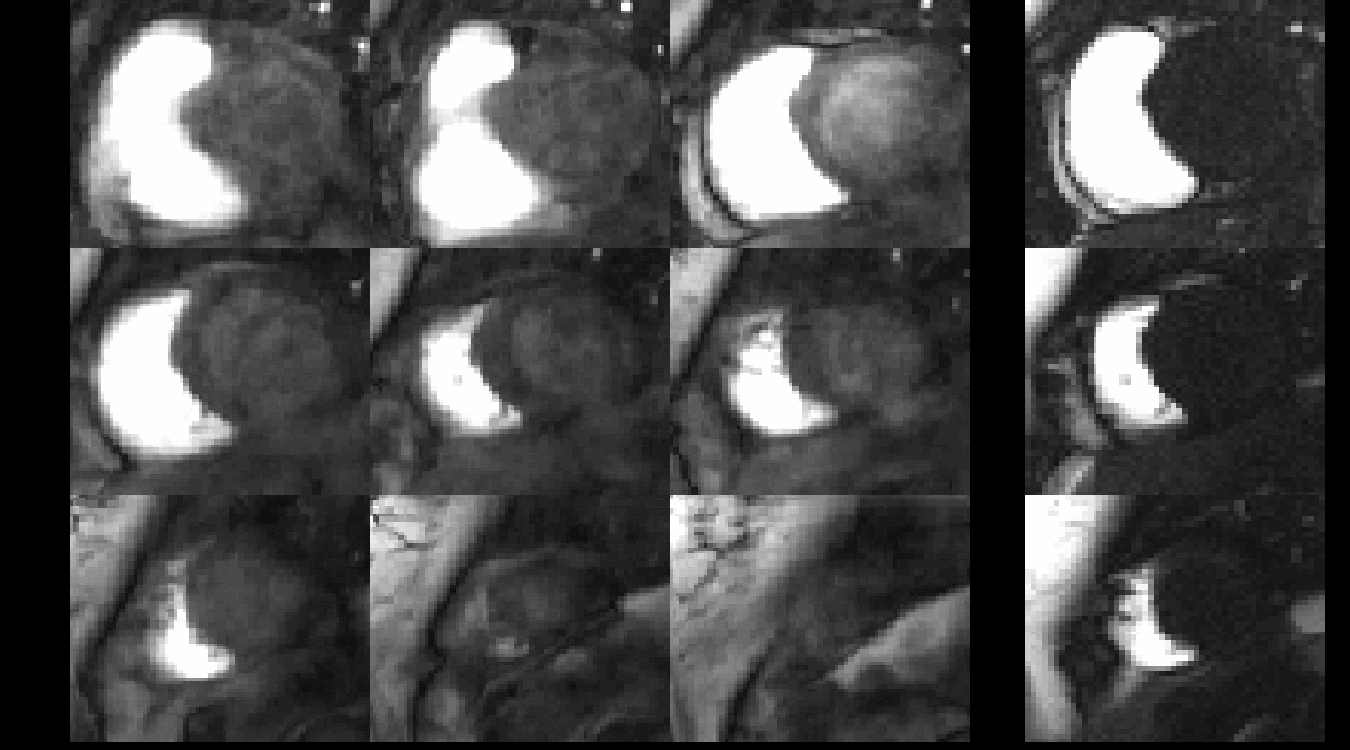

Supplement: Supplementary file 5 — Video S4 Video of perfusion images acquired in the patient presented in Supporting Information Figure S1. [file MRM-88-663-s004.gif]
